# Addition of navitoclax to ruxolitinib for patients with myelofibrosis with progression or suboptimal response

**DOI:** 10.1016/j.bneo.2024.100056

**Published:** 2024-11-02

**Authors:** Naveen Pemmaraju, Tim C.P. Somervaille, Francesca Palandri, Claire Harrison, Rami S. Komrokji, Andrew Perkins, Rosa M. Ayala Diaz, David Lavie, Akihiro Tomita, Yang Feng, Qin Qin, Jason Harb, Akshanth R. Polepally, Jalaja Potluri, Jacqueline S. Garcia

**Affiliations:** 1 Department of Leukemia, The University of Texas MD Anderson Cancer Center, Houston, TX; 2 Department of Haematology, The Christie National Health Service Foundation Trust, Manchester, United Kingdom; 3 Cancer Research UK Manchester Institute, The University of Manchester, Manchester, United Kingdom; 4 Department of Haematology, Istituto di Ricovero e Cura a Carattere Scientifico Azienda Ospedaliero-Universitaria di Bologna, Istituto di Ematologia “Seràgnoli,” Bologna, Italy; 5 Malignant Hematology Department, Guy’s and St Thomas’ NHS Foundation Trust, London, United Kingdom; 6 Moffitt Cancer Center, Tampa, FL; 7 The Alfred Hospital and The Australian Centre for Blood Diseases, Monash University, Melbourne, Australia; 8 Hospital Universitario 12 de Octubre, I+12, Complutense University, Madrid, Spain; 9 Department of Hematology, Hadassah-Hebrew University Medical Center, Jerusalem, Israel; 10 Department of Hematology, Fujita Health University School of Medicine, Toyoake, Japan; 11 Clinical Development Oncology, AbbVie Inc, North Chicago, IL; 12 Department of Oncology, Dana-Farber Cancer Institute, Boston, MA

## Abstract

Navitoclax (oral B-cell lymphoma-2 family protein inhibitor induces apoptosis of malignant cells in myelofibrosis (MF). We present pooled cohort 1 results from the phase 2 REFINE trial, which evaluated navitoclax plus ruxolitinib (NAV+RUX) for patients with relapsed/refractory MF with suboptimal response to RUX (≥10 mg twice daily stable dose for ≥12 weeks [cohort 1a] or ≥24 weeks [cohort 1b]). Cohort 1a received add-on NAV 50 mg/d, with escalation to ≤300 mg if platelet count was ≥75 × 10^9^/L. Cohort 1b received NAV 100 or 200 mg/d if platelet count was ≤150 or >150 × 10^9^/L, respectively. The primary end point was spleen volume reduction of ≥35% (SVR_35_) at week 24. Secondary end points included ≥50% total symptoms score (TSS_50_) reduction at week 24, bone marrow fibrosis (BMF) grade changes, anemia response, and safety. In total, 125 patients received ≥1 dose of NAV+RUX. With median follow-up of 21 months, SVR_35_ rate was 23% at week 24 and 39% at any time on study (median duration: 11 months). TSS_50_ rate was 24% at week 24 and 46% at any time on study. BMF improved by ≥1 grade, any time on study, in 39% of patients. Anemia responses were achieved in 23% of patients. Median overall and progression-free survival were 52.3 and 22.1 months, respectively. No new safety signals were observed. The most common adverse event was thrombocytopenia without clinically significant bleeding. NAV+RUX was tolerable and demonstrated early improvement in disease modification parameters in this difficult-to-treat population. This trial was registered at www.ClinicalTrials.gov as #NCT03222609.

## Key Points

• Adding navitoclax to ongoing ruxolitinib demonstrates durable responses and potential disease modification in relapsed/refractory MF.
• Thrombocytopenia is the most common adverse event and is manageable and reversible with dose reduction as necessary.

## Visual Abstract

**Figure:**
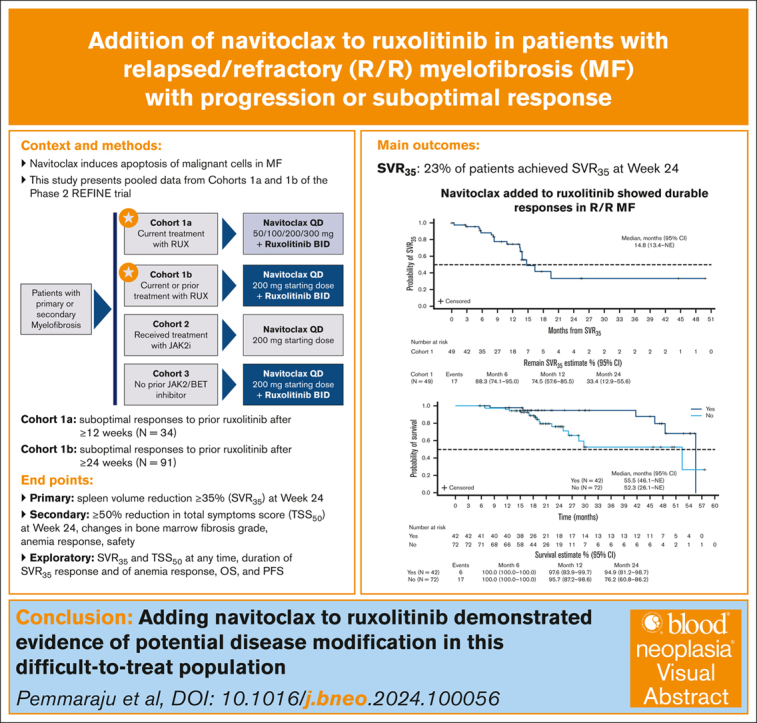

## Introduction

Myelofibrosis (MF) is a myeloproliferative neoplasm (MPN) primarily driven by constitutive activation of the Janus kinase/signal transducers and activators of transcription (JAK/STAT) signaling pathway.^1^^,^^2^ Clinical manifestations include bone marrow fibrosis (BMF), splenomegaly, anemia, and debilitating constitutional symptoms that significantly compromise patient quality of life^3^^,^^4^ and reduce survival.^5^^,^^6^

JAK inhibitors (JAKis), including ruxolitinib (JAK1/2i), fedratinib (JAK2i), pacritinib (JAK2i), and momelotinib (JAK1/2i), are currently the only approved therapies for patients with MF^7^, ^8^, ^9^, ^10^; but they rely on spleen and symptom relief as key response criteria rather than modification of the underlying disease.^11^ Despite improvements in quality of life, up to 73.3% of patients discontinue ruxolitinib by 5 years.^12^^,^^13^ Patients with MF with suboptimal responses to, or progression on, JAKis represent a population of unmet need given the lack of approved therapeutic strategies in this setting.^14^^,^^15^

Owing to constitutive activation of the JAK/STAT pathway, the antiapoptotic B-cell lymphoma–2 (BCL-2) family protein, BCL-extra large (BCL-X_L_), is commonly overexpressed in MPNs, including MF; therefore, BCL-X_L_ is a potentially promising therapeutic target in these diseases.^16^, ^17^, ^18^ Navitoclax (ABT-263), an orally bioavailable, small-molecule BCL-2 homology 3 mimetic, binds with high affinity to prosurvival BCL-2 family proteins (BCL-X_L_, BCL-2, and BCL-W), disrupting interactions with proapoptotic factors, and promoting apoptosis of malignant cells.^19^ In preclinical models, synergistic cell killing between navitoclax and JAK1/2 inhibitors, including ruxolitinib, has been described,^16^ and the addition of navitoclax substantially enhances ruxolitinib activity in ruxolitinib-resistant cells, resulting in enhanced cell death.^20^

REFINE is, to our knowledge, the first clinical trial examining the efficacy and safety of adding navitoclax to ruxolitinib for patients with primary or secondary MF with disease progression or suboptimal response to ruxolitinib monotherapy. We previously reported the safety and efficacy of navitoclax plus ruxolitinib for patients with relapsed/refractory (R/R) MF and suboptimal responses to ruxolitinib after ≥12 weeks (cohort 1a, n = 34). This treatment combination resulted in durable spleen volume reduction of ≥35% (SVR_35_), improvements in BMF, ≥50% reduction in total symptoms score (TSS_50_), and an anemia response. The most common adverse event (AE) was thrombocytopenia that was generally reversible and without clinically significant bleeding.^21^ Cohort 1b comprises a larger population of 91 patients with R/R MF and suboptimal responses to ruxolitinib after ≥24 weeks and is a more difficult-to-treat population than cohort 1a, with patients presenting with a longer time since diagnosis, longer time on ruxolitinib, larger spleen volume, and higher Dynamic International Prognostic Scoring System (DIPSS) risk at baseline. This pooled analysis evaluates the efficacy and safety of navitoclax plus ruxolitinib in 125 patients in REFINE cohorts 1a and 1b.

## Methods

### Study design and patients

**Figure 1.**
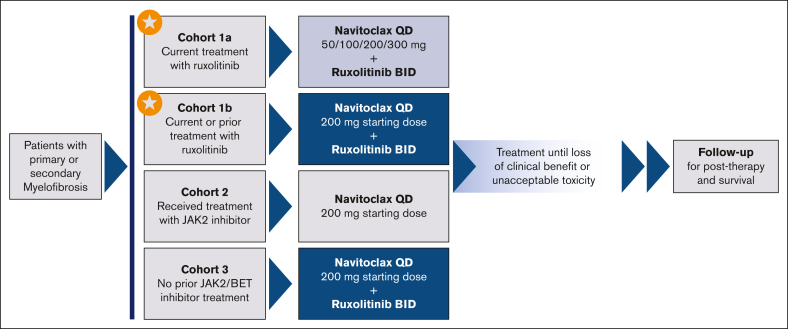
**REFINE study schema.** Schematic diagram shows the distinction between the 3 cohorts included in the REFINE study. Starred cohorts are included in this analysis. BET, bromodomain and extraterminal; BID, twice daily; QD, once daily.

The REFINE study is a global phase 2 multicenter nonrandomized open-label trial designed to evaluate the tolerability and efficacy of navitoclax as monotherapy (cohort 2 [prior JAK2i]) or in combination with ruxolitinib (cohort 1 [current/prior ruxolitinib] and cohort 3 [JAK2i naïve]) for patients with primary or secondary MF (Figure 1). Here, we present the pooled results from cohort 1, which was conducted across 57 global study sites and enrolled patients between 31 October2017 and 10 April 2019 (ClinicalTrials.gov identifier: NCT03222609).

Patients in cohort 1 were to have suboptimal responses to ruxolitinib on a stable dose of ≥10 mg twice a day after ≥12 weeks (cohort 1a) or ≥24 weeks (cohort 1b) of treatment; for full details on inclusion criteria, see supplemental Methods. Patients with progression of disease on ruxolitinib ≥28 days to <24 weeks could also be enrolled in cohort 1b per inclusion criteria. Disease progression while on ruxolitinib was defined by (1) in patients with no evidence of splenomegaly before the initiation of ruxolitinib: a new splenomegaly palpated to ≥5 cm below the left costal margin; (2) in patients with measurable spleen distance 5 to 10 cm or >10 cm before the initiation of ruxolitinib: a ≥100% or ≥50% increase in the palpable distance below the left costal margin, respectively; or (3) in patients with a spleen volume assessment before the initiation of ruxolitinib: a spleen volume increase of ≥25%. In cohort 1a, patients received a starting dose of 50 mg/d navitoclax, with stepwise increase to ≤300 mg if platelet count was ≥75 × 10^9^/L. In cohort 1b, patients started with 100 mg daily dose of navitoclax if platelet count was ≤150 × 10^9^/L, or 200 mg if platelet count was >150 × 10^9^/L; 300 mg navitoclax dose was permitted at week 24 for patients with suboptimal spleen response with continued ruxolitinib. The relative dose intensity of navitoclax and ruxolitinib was calculated for each individual and summarized using study drug dosing information (supplemental Methods).

Detailed methods and eligibility have been described previously.^21^ Patients were treated until disease progression, unacceptable toxicity, or withdrawal of consent. All patients were followed-up for safety for 30 days after treatment discontinuation. Those who discontinued for reasons other than disease progression were followed-up for ∼12 weeks until disease progression or initiation of another treatment for MF. Patients who discontinued navitoclax but continued ruxolitinib monotherapy as poststudy treatment were considered discontinued from both study drugs. Patients were followed-up every 6 months for survival and will continue to be followed-up for up to 5 years after treatment discontinuation.

The study protocol, informed consent, and all other forms were approved by an independent ethics committee or institutional review board. The study was conducted in accordance with the protocol, International Conference on Harmonization guidelines,^22^ good clinical practice guidelines, and ethical principles from the Declaration of Helsinki. All patients provided written informed consent to participate in this trial.

### End points and assessments

The primary efficacy end point was SVR_35_ at week 24 measured by magnetic resonance imaging or computed tomography with central review. Spleen volume was evaluated at screening and at weeks 12, 24, 36, 48, 72, and 96. Secondary efficacy end points included TSS_50_ assessed by the myelofibrosis symptom assessment form version 4.0^23^^,^^24^ at week 24; anemia response rate per modified International Working Group for Myeloproliferative Neoplasms Research and Treatment^4^^,^^25^; and change in BMF grade, locally reviewed by institutional pathologists according to the European consensus grading system.^26^ Exploratory end points included SVR_35_ and TSS_50_ at any time on study, duration of SVR_35_ response, duration of anemia response, overall survival (OS), and progression-free survival (PFS). PFS included death and progression, including leukemic transformation confirmed by a bone marrow blast count of ≥20%. Safety was evaluated throughout the study and ≤30 days after the last dose of study treatment. AEs and laboratory evaluations were assessed in accordance with National Cancer Institute Common Terminology Criteria for Adverse Events, version 4.03.^27^

### Statistical methods

The sample size calculation for cohort 1a was previously described.^21^ For cohort 1b, a sample size of 70 was estimated to provide assurance that the true SVR_35_ rate at week 24 would be within ∼12.2% of the observed rate with 95% confidence. Furthermore, if the true probability of experiencing a serious AE due to study treatment in cohort 1b was 10%, then the probability of observing at least 1 serious AE in 70 patients was >99%, which was considered adequate. Statistical analyses were conducted using SAS version 9.4 (SAS Institute, Inc, Cary, NC), as outlined by Harrison et al.^21^ See supplemental Methods for details on statistical methods.

## Results

### Patient demographics and baseline characteristics

At the data cutoff (16 January 2023), 125 patients with MF had received ≥1 dose of navitoclax plus ruxolitinib (cohort 1a, n = 34; cohort 1b, n = 91). Baseline demographics and characteristics of the pooled cohort 1 are shown in Table 1. Median age of pooled cohort 1 was 69 years (range, 34-86), and 64% of patients were male. Median duration of prior ruxolitinib exposure for MF treatment was 95 weeks (range, 8-464). At study entry, 84 (72%) patients had *JAK2* mutations, 26 (22%) had calreticulin (*CALR)* mutations, 3 (3%) had *MPL* mutations, and 3 (3%) were triple negative. In total, 71 (61%) of patients had high molecular risk (HMR) mutations.

**Table 1.** **Baseline demographics and disease characteristics**

Characteristic	Cohorts 1a and 1b (N = 125)	Cohort 1a^21^ (N = 34)
Age (y), median (range)	69 (34-86)	68 (42-86)
**Sex, n (%)**		
Male	80 (64)	23 (68)
Female	45 (36)	11 (32)
**Race, n (%)**		
White	108 (86)	32 (94)
Black or African American	2 (2)	2 (6)
Asian	15 (12)	0 (0)
**ECOG performance status, n (%)**		
0	52 (42)	16 (47)
1	67 (54)	18 (53)
2	6 (5)	0 (0)
Prior lines of therapy for MF, median (range)	1 (1-6)	1 (1-6)
Baseline platelet count (×10^9^/L), median (range)	201 (75-1469)	201 (98-706)
Baseline spleen volume (cm^3^), median (range)	2128 (448∗-6219)	1695 (466-5047)
Baseline white blood cell count (×10^9^/L), median (range)	15 (2-205)	19 (5-205)
**Baseline hemoglobin (g/dL), median (range)**	10 (5-15)	11 (7-15)
Baseline hemoglobin ≥8 to <10 g/dL, n (%)	34 (27)	6 (18)
**Transfusion status, n (%)**		
Dependent	6 (13)	4 (12)
Independent	109 (87)	30 (88)
Transfusion required ≤12 wk before study entry	32 (26)	6 (18)
Baseline TSS, median (range)	17 (0-58)	14 (0-35)
Time from diagnosis to study entry (mo), median (range)	43 (1-338)	28 (1-199)
Duration of prior ruxolitinib exposure for MF treatment (wk), median (range)	95 (8-464)	70 (13-391)
**DIPSS risk at study entry, n (%)**		
Intermediate-1	36 (29)	17 (50)
Intermediate-2	71 (57)	12 (35)
High	17 (14)	4 (12)
***JAK2* status at study entry, n (%)**		
Detected	84 (72)	25 (78)
***CALR* status at study entry, n (%)**		
Detected	26 (22)	7 (22)
***MPL* status at study entry, n (%)**		
Detected	3 (3)	0 (0)
**HMR status at study entry**†**, n (%)**		
Detected	71 (61)	19 (59)

Data are n (%) unless stated otherwise.

*CALR*, calreticulin; ECOG, Eastern Cooperative Oncology Group.

∗ One patient with SVR of <450 cm^3^ by central review was included because of a SVR of ≥450 cm^3^ by local review.

† Defined as mutations in *ASXL1, SRSF2, EZH2, U2AF1 (Q157), IDH1*, or *1DH2*.

Patients received navitoclax for a median of 70 weeks (range, 3-250). As of data cutoff, 77 (62%) patients had discontinued navitoclax and ruxolitinib, primarily because of AEs (navitoclax, n = 28 [22%]; ruxolitinib, n = 12 [10%]) and progressive disease (n = 24 [19%]). Detailed reasons for study drug discontinuations are provided in supplemental Figure 1. Overall, 35 (28%) patients discontinued the study; 29 (23%) patients discontinued because of deaths, 5 (4%) patients withdrew consent, and 1 (<1%) patient was lost to follow-up (supplemental Figure 1). Eight (6.4%) patients went on to receive poststudy stem cell transplant whereas 5 patients exhibited ≥20% blasts in the bone marrow or peripheral blood, indicating transformation to acute myeloid leukemia.

### Efficacy assessments

**Figure 2.**
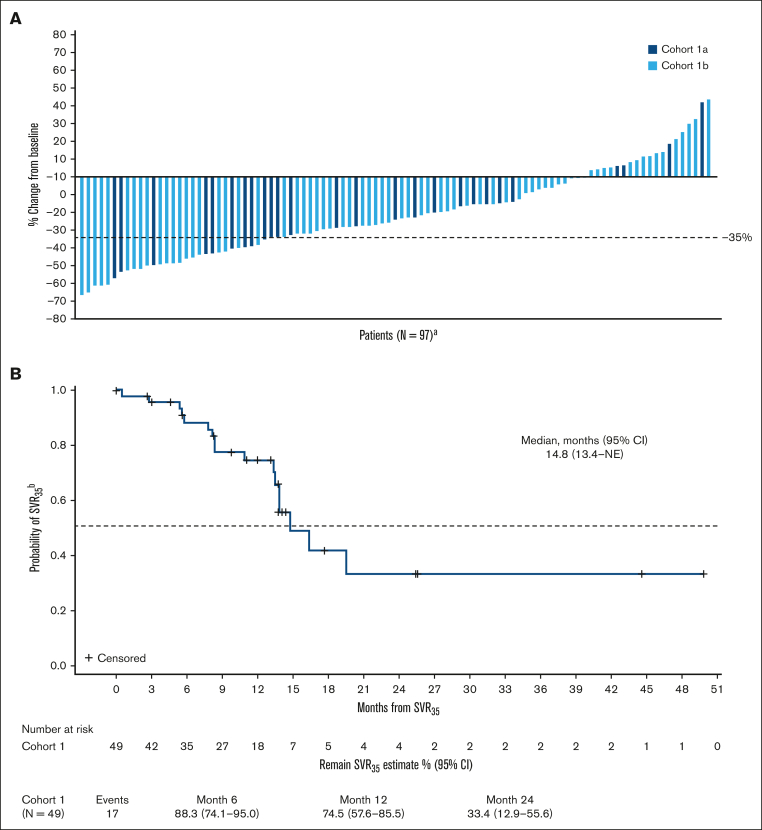
**Changes in spleen volume over time.** (A) Waterfall plot shows percentage change from baseline in spleen volume at week 24 for individual patients in cohorts 1a and 1b; (B) Kaplan-Meier curve depicts the probability of maintaining SVR_35_ for patients who achieved it at any time on study. ^a^N, number of patients with nonmissing percent change in spleen volume from baseline at week 24; ^b^from time that it was achieved.

**Figure 3.**
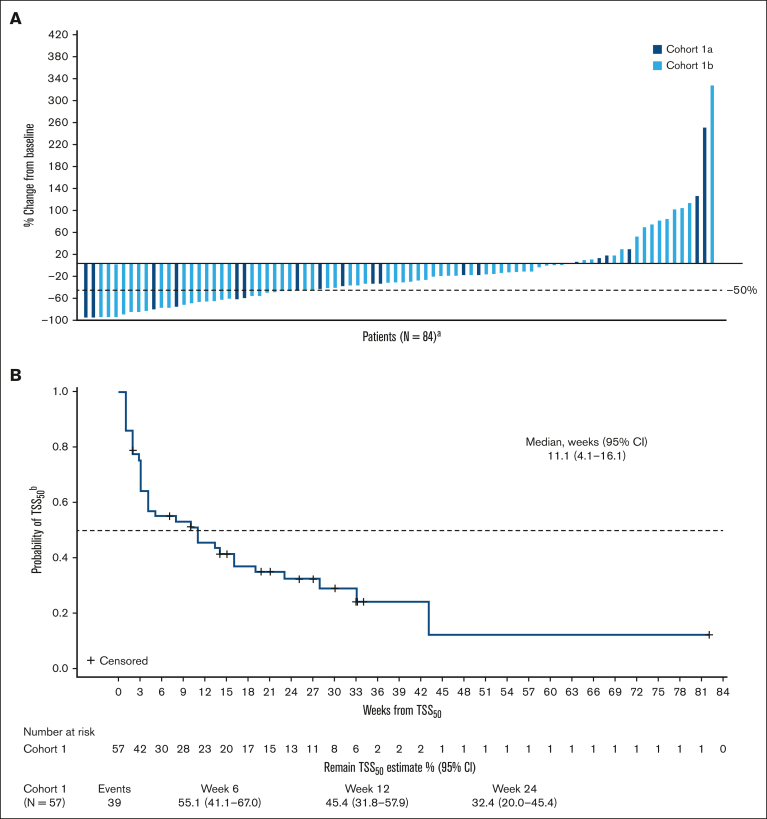
**Changes in total symptom score over time.** (A) Waterfall plot shows percentage change from baseline in TSS at week 24 for individual patients in cohorts 1a and 1b; (B) Kaplan-Meier curve depicts the probability of maintaining TSS_50_ for patients who achieved it at any time on study. ^a^N, number of patients with nonmissing percent change in TSS from baseline at week 24; ^b^from time that it was achieved.

Patients were followed-up for a median of 21 months (range, 1.6-58.7). SVR_35_ was achieved in 29 (23%) patients (95% confidence interval [CI], 16.1-31.6) at week 24 (supplemental Table 1; Figure 2A) and in 49 (39%) patients (95% CI, 30.6-48.3) at any time on study (supplemental Table 1; supplemental Figure 2). SVR_35_ at week 24 in patients with refractory or stable disease and relapsed MF were 22% (95% CI, 13.3-33.6) and 24% (95% CI, 8.2-47.2), respectively. The median time to first SVR_35_ was 13 weeks (range, 11-75), and SVR_35_ was observed as late as week 96 (n = 4; 3%). The estimated median duration of SVR_35_ on study was 14.8 months (Figure 2B). Within high-risk groups known to confer poor prognosis, SVR_35_ at any time on study was achieved in 42% of patients (35/83) aged ≥65 years, 38% of patients (27/71) with intermediate-2 DIPSS score, 47% of patients (8/17) with high DIPSS score, and 35% of patients (25/71) with HMR mutations (supplemental Table 1). TSS_50_ was achieved in 30 (24%) patients at week 24 (supplemental Table 1; Figure 3A) and 57 patients (46%) at any time on study (supplemental Table 1; supplemental Figure 3). Median time to first TSS_50_ was 5.3 months (range, 0.3-29). The estimated median duration of TSS_50_ was 11 weeks (Figure 3B). Within high-risk groups, TSS_50_ at any time on study was achieved in 46% of patients aged ≥65 years, 55% of patients with intermediate-2 DIPSS score, 18% of patients with high DIPSS score, and 45% of patients with HMR mutations (supplemental Table 1). The average daily dose of navitoclax was 162 and 141 mg for patients who achieved and did not achieve SVR_35_, respectively, and 149 and 145 mg for patients who achieved and did not achieve TSS_50_, respectively. The average daily dose of ruxolitinib was 22 mg and 23 mg for patients who achieved and did not achieve SVR_35_, respectively, and 21 and 23 mg for patients who achieved and did not achieve TSS_50_, respectively.

Of 110 patients with matched baseline and postbaseline data, BMF improved by ≥1 grade in 42 (39%) patients at any time on study (supplemental Table 1); 15 of 99 (15%) at week 12, 19 of 80 (24%) at week 24, 25 of 64 (39%) at week 48, 6 of 15 (40%) at week 96. Of 42 patients with improved BMF by ≥1 grade at any time, 32 of 110 (29%) patients improved by 1 grade, and 8 of 110 (7%) patients improved by 2 grades; 2 of 110 (2%) patients improved by 3 grades, the remaining 68 of 110 (62%) patients had either unchanged (59/110; 54%) or worsened (9/110; 8%) BMF grade from baseline, with 69 of 110 (63%) patients having grade 3 BMF at baseline. Spleen volume reduced by 32% and TSS improved by 6.5 points from baseline at week 24 in patients with improved BMF by ≥1 grade. Among patients with and without a BMF grade reduction of ≥1, respectively, SVR_35_ was achieved in 60% (95% CI, 43.3-74.4) and 29% (95% CI, 19.4-40.4) of patients at any time, and SVR_50_ was achieved in 26% (95% CI, 9.2-51.2) and 10% (95% CI, 4.2-19.8) of patients at week 24.

### Time-to-event end points

**Figure 4.**
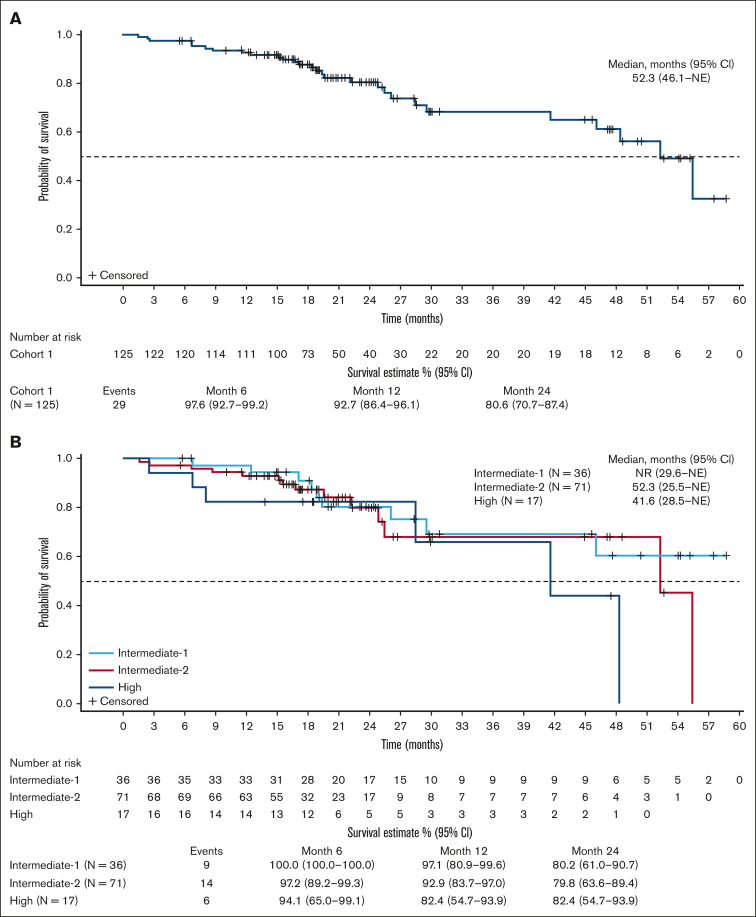
**OS after treatment with navitoclax and ruxolitinib.** (A) Kaplan-Meier curve of OS for pooled cohort 1; (B) Kaplan-Meier curve of OS by DIPSS risk for pooled cohort 1. NR, not reached.

Median OS was 52.3 months (95% CI, 46.1 to not estimable [NE]) for all patients (Figure 4A); and was not reached, 52.3 months, and 41.6 months for intermediate-1, intermediate-2, and high DIPSS-risk patients, respectively (Figure 4B). With a median follow-up of 21 months for pooled cohort 1 (cohort 1a, 50 months; cohort 1b, 18 months), the estimated OS at 24 months was 81% (95% CI, 71-87). Of patients with improved BMF by ≥1 grade at any time on study (n = 42), estimated OS was 55.5 months (95% CI, 46.1 to NE; supplemental Figure 4). Median PFS for all patients in cohort 1 was 22.1 months (95% CI, 16.9-28.5; supplemental Figure 5), and the estimated PFS at 24 months was 43% (95% CI, 30-56).

### Anemia responses

**Figure 5.**
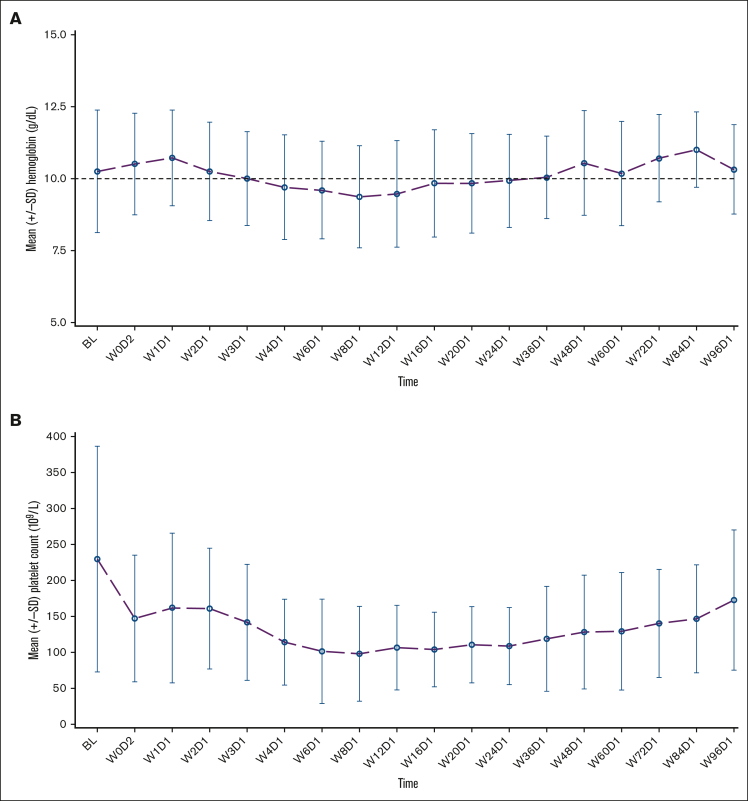
**Mean hemoglobin levels and platelet count over time for all treated patients.** (A) Graph shows mean (with standard deviation) hemoglobin level by visit for pooled cohort 1; (B) graph shows mean (with standard deviation) platelet count by visit for pooled cohort 1. BL, baseline; D, day; SD, standard deviation; W, week.

Anemia responses per modified International Working Group for Myeloproliferative Neoplasms Research and Treatment criteria were achieved in 23% (n = 14) of 61 evaluable patients (95% CI, 13.2-35.5) at any time on study. This included 10 (22%) of 45 patients with hemoglobin of <10 g/dL who were transfusion independent (TI) at baseline (hemoglobin improvement of ≥2 g/dL), and 4 (25%) of 16 patients who were transfusion dependent at baseline (defined as transfusion of at least 6 units of packed red blood cells in the 12 weeks before study enrollment, for a hemoglobin level of <8.5 g/dL, without bleeding or treatment-induced anemia). The estimated median duration of anemia response was 11.8 months (95% CI, 0.7 to NE) among responders who were TI with improved hemoglobin, and 16.1 months (range, 5.4-29.5) among patients who were transfusion dependent at baseline and achieved TI. The mean hemoglobin level was <10 g/dL at weeks 12 and 24, and increased to >10 g/dL at week 48 after the initiation of combined treatment (Figure 5A).

### Safety

Median duration of exposure to navitoclax was 70 weeks (range, 3-250) and to ruxolitinib was 70 weeks (range, 3-241). The most common AEs of any grade included thrombocytopenia (86%), diarrhea (59%), anemia (43%), nausea (31%), fatigue (30%), and neutropenia (26%); the most common grade ≥3 AEs included thrombocytopenia (grade 3, 51%; grade 4, 14%), anemia (all grade 3, 34%), and neutropenia (grade 3, 18%; grade 4, 7%; Table 2). Serious treatment-emergent AEs (TEAEs) (>2% of patients) were pneumonia (n = 7; 6%), COVID-19 infection (n = 5; 4%), multiple organ dysfunction syndrome (n = 4, 3%), febrile neutropenia (n = 3, 2%), pyrexia (n = 3, 2%), and cerebrovascular accident (all n = 3, 2%). Non–COVID-19 infection rates were low (n = 11, 9%). Any-grade bleeding events were reported in 34% of patients without clinically significant sequelae. There was 1 event of unrelated grade 3 hemorrhage, which led to treatment discontinuation. Thrombocytopenia associated with the combination of navitoclax and ruxolitinib was manageable and reversible with dose reduction/interruption of navitoclax and/or ruxolitinib. The mean platelet count was ≥100 × 10^9^/L at 8, 12, and 24 weeks after the initiation of combined treatment (Figure 5B). The most common any-grade gastrointestinal (GI) events were diarrhea (59%, n = 74), nausea (31%, n = 39), abdominal pain (18%, n = 22), constipation (12%, n = 15), and vomiting (11%, n = 14). Diarrhea was the most common grade ≥3 GI event (5%, n = 6).

**Table 2.** **Overview of safety**

Safety characteristic	Cohorts 1a and 1b (N = 125)
Any TEAE	125 (100)
**Most common TEAEs (≥10% patients) of any grade**	
Thrombocytopenia	108 (86)
Diarrhea	74 (59)
Anemia	54 (43)
Nausea	39 (31)
Fatigue	37 (30)
Neutropenia	33 (26)
COVID-19 infection	25 (20)
Abdominal pain	22 (18)
Arthralgia	20 (16)
Contusion	20 (16)
Dizziness	19 (15)
Headache	19 (15)
Hyperuricemia	19 (15)
Alanine aminotransferase increased	17 (14)
White blood cell count decreased	17 (14)
Constipation	15 (12)
Edema peripheral	15 (12)
Pyrexia	15 (12)
Decreased appetite	14 (11)
Urinary tract infection	14 (11)
Vomiting	14 (11)
Aspartate aminotransferase increased	13 (10)
Blood bilirubin increased	13 (10)
Dyspnea	13 (10)
Upper respiratory tract infection	13 (10)
Any grade ≥3 AE	101 (81)
**Most common grade ≥3 TEAEs (≥10% patients)**	
Thrombocytopenia	66 (53)
Grade 3	64 (51)
Grade 4	18 (14)
Anemia (all grade 3)	43 (34)
Neutropenia	24 (19)
Grade 3	23 (18)
Grade 4	9 (7)
Serious TEAEs	48 (38)
TEAEs leading to navitoclax discontinuation∗	33 (26)
**Key TEAEs leading to navitoclax discontinuation**†	
Thrombocytopenia	9 (7)
Diarrhea	4 (3)
Neuroendocrine carcinoma of the skin	2 (2)
TEAEs leading to ruxolitinib discontinuation∗	18 (14)
**Key TEAEs leading to ruxolitinib discontinuation**†	
Thrombocytopenia	4 (3)
TEAE leading to navitoclax interruption	84 (67)
TEAE leading to ruxolitinib interruption	57 (46)
TEAE leading to navitoclax reduction	90 (72)
TEAE leading to ruxolitinib reduction	77 (62)
TEAE leading to death‡	11 (9)
**All deaths**	29 (23)
Deaths occurring ≤30 d after last dose of navitoclax	11 (9)
Deaths occurring >30 d after last dose of navitoclax	18 (14)

All data are n (%).

∗ Patients that discontinued the study drug could remain on the study for poststudy treatment follow-up and survival follow-up. Safety assessments were conducted for up to 30 days after study drug discontinuation.

† TEAEs reported as the primary reason for treatment discontinuation (full details in supplemental Figure 1).

‡ Multiple organ dysfunction (n = 4), COVID-19 (n = 2), pneumonia (n = 2), cardiac arrest (n = 1), infection (n = 1), septic shock (n = 1), and fall (n = 1); patients may have experienced >1 TEAE contributing to death.

Relative median (range) dose intensity was 72% (range, 13-133) for navitoclax and 100% (range, 34-250) for maintaining the minimum protocol-specified stable starting ruxolitinib dose of 10 mg twice a day. For more details, see supplemental Results. Navitoclax dose reduction, interruption, and discontinuation because of TEAEs occurred in 90 (72%), 84 (67%), and 33 (26%) patients, respectively (Table 2). Thrombocytopenia was the most common TEAE leading to these navitoclax dose modifications occurring in 60% (reduction), 50% (interruption), and 7% (navitoclax discontinuation) of patients. Bleeding was not a reported reason for dose reduction. Median time to onset of any-grade thrombocytopenia was 22 days (range, 1-589). Ruxolitinib dose reduction, interruption, and discontinuation because of TEAEs occurred in 77 (62%), 57 (46%), and 18 (14%) patients, respectively (Table 2). Similarly, thrombocytopenia was also the most common TEAE leading to these ruxolitinib dose modifications, occurring in 54% (reduction), 34% (interruption), and 3% (discontinuation) of patients. Of 96 patients who received navitoclax up to 200 mg, dose was reduced in 73 patients and interrupted in 58 patients, whereas ruxolitinib dose was reduced in 58 patients and interrupted in 38 patients. In total, 4 (3%) patients discontinued navitoclax within 20 to 262 days and 1 (1%) patient discontinued ruxolitinib after 20 days, because of decreases in platelet counts. In addition, 5% (n = 6) and 2% (n = 2) of patients discontinued navitoclax and ruxolitinib because of GI events, respectively. Of 29 (23%) patients who died, 11 (9%) died ≤30 days after the last dose of navitoclax; no deaths were deemed related to navitoclax or ruxolitinib.

## Discussion

In cohort 1 of this phase 2 open-label, multicenter REFINE study, navitoclax plus ruxolitinib demonstrated durable clinical benefit and a generally manageable safety profile in patients with R/R MF with prolonged prior ruxolitinib exposure. SVR_35_ was achieved in 23% of patients at week 24 and in 39% at any time, with an estimated median duration of 11 months. SVR_35_ at any time correlated strongly with reduction in BMF grade. Encouraging SVR_35_ rates were observed despite baseline HMR mutation status, known to confer poor prognosis.^28^^,^^29^ Other efficacy end points of TSS_50_, BMF, anemia responses, OS, and PFS also demonstrated promising outcomes. The combination of navitoclax and ruxolitinib was tolerable; thrombocytopenia (without bleeding) was expected, common, and generally reversible with protocol-guided dose adjustments. Navitoclax was successfully restarted in 85% of patients requiring dose interruption for thrombocytopenia. This is, to our knowledge, the first trial to investigate and show clinically meaningful and durable responses with navitoclax and ruxolitinib in this difficult-to-treat population.

Findings from pooled cohort 1 validate and enhance robustness of previously published observations from cohort 1a.^21^ Notably, cohort 1b includes a more challenging-to-treat population compared with cohort 1a, including a higher proportion of patients with intermediate-2 DIPSS risk, larger median baseline spleen volume, longer time from diagnosis of MF, and longer time on ruxolitinib monotherapy. These are clinically relevant because cohort 1b was considerably larger (n = 91 cohort 1b vs n = 34 cohort 1a). Similar results were observed between pooled cohort 1 and cohort 1b for SVR_35_ rates and TSS_50_, at week 24 and at any time on study; and BMF improvement from baseline by ≥1 grade at any time on study. The observation of SVR_35_ achievement and BMF improvement as late as week 96 in pooled cohort 1 supports the suggestion that this combination may positively affect disease modification, with responses becoming evident beyond the initial 24 weeks. Anemia response is a significant medical need for patients with MPNs.^30^ Recent observations in patients with MF have reported anemia response (transfusion independence) in 19% to 22% of patients treated with pacritinib,^31^ and 31 to 43% treated with momelotinib.^32^^,^^33^ Promising anemia response rates were achieved with navitoclax and ruxolitinib in both pooled cohort 1 and cohort 1a, with responses reported in 23% and 36% of patients, respectively. Median OS for pooled cohort 1 was 52.3 months and was not reached in cohort 1a.^21^

Deep and durable responses, as well as evidence of disease modification have been reported in additional analyses of REFINE.^34^^,^^35^ Post hoc analysis of molecular biomarkers in REFINE cohort 1a reported clinically meaningful responses in SVR_35_ (31%), irrespective of HMR mutation status. In the HMR group, TSS_50_ was achieved in 36% of patients and BMF improved by ≥1 grade in 39% of patients at week 24; patients with improvements in fibrosis had better median OS than those without improvement, implying disease modification.^34^ Efficacy responses, including the correlation between SVR and BMF grade reduction, reductions in variant allele frequency of MPN driver mutations in patients with HMR mutations (supplemental Table 1), and OS data reported in this study provide additional support for disease modification by the combination.

In REFINE cohort 1, robust and promising efficacy outcomes were demonstrated in patients with R/R MF, for whom outcomes are typically poor after ruxolitinib discontinuation and additional treatment is limited.^36^^,^^37^ Several previous studies have reported on treatment with ruxolitinib as monotherapy,^13^^,^^38^^,^^39^ fedratinib after ruxolitinib,^40^ momelotinib after ruxolitinib,^32^ and pacritinib after JAKis,^31^ with SVR_35_ at week 24 ranging from 7% to 42%.^13^^,^^31^^,^^32^^,^^38^ The SVR_35_ rate in our population of patients with suboptimal response to prior ruxolitinb monotherapy is encouraging given the lower rates observed in previous studies.^31^^,^^32^ The OS observed in pooled cohort 1 supports earlier suggestions that this combination may increase OS compared with conventional or targeted therapies after ruxolitinib discontinuation, with which median OS was up to 14 months.^36^^,^^37^^,^^41^ The suggested synergistic mechanism of action between navitoclax and ruxolitinib in treating R/R MF^16^ appears promising based on these data. The enhanced efficacy of this treatment combination may provide a more effective therapeutic approach and patient benefit in this challenging patient population.

The safety summary of the pooled cohort 1 was similar to cohort 1a, with similar rates of dose modifications. In both cohorts, the most common AE of thrombocytopenia was manageable and reversible with dose reduction as necessary, and no new safety signals reported.^21^ Types of safety events of cohort 1 were broadly similar to studies of ruxolitinib monotherapy, with some differences in AE rates. Although thrombocytopenia occurred more frequently with the combination than the rates reported for ruxolitinib alone,^13^^,^^38^ it was mostly reversible after dose adjustments, indicating that navitoclax combinations can be tolerated over a longer duration, with cytopenias expected but generally manageable.^30^

Key strengths of this pooled analysis include the moderate sample size of patients with a longer duration of ruxolitinib without pretreatment washout, consistent with real-world treatment, and the long follow-up duration. The novel finding of durable OS in this larger, higher-risk population (including in patients with intermediate-2 and high DIPSS risk) implies disease modification with navitoclax plus ruxolitinib. Although findings suggest these patients may derive long-term efficacy benefit from navitoclax plus ruxolitinib, with improved survival and tolerability, a definitive interpretation is limited given the open-label study design and lack of comparator arm. Also, evaluation of BMF was by local pathologists. Further studies are warranted to support these promising observations. Navitoclax is currently being evaluated in a phase 3 trial in this population; TRANSFORM-2 (ClinicalTrials.gov identifier: NCT04468984).^42^ Further studies investigating predictive biomarkers of response to navitoclax are underway.

Navitoclax plus ruxolitinib demonstrated clinically meaningful efficacy and symptom improvement in patients with R/R MF and suboptimal response to prior ruxolitinib, validating previous observations in a smaller cohort.^21^ This combination was tolerable and showed a generally manageable safety profile; the most common and expected AE of thrombocytopenia was well managed with dose modifications, without any clinical sequelae. These phase 2 data support the ongoing phase 3 TRANSFORM-2 trial.

Conflict-of-interest disclosure: N.P. reports consulting or advisory role with 10.13039/100006436Celgene, Stemline Therapeutics, 10.13039/100017655Incyte, 10.13039/100004336Novartis, Mustang Bio, 10.13039/100016545Roche Diagnostics, and LFB; honoraria from 10.13039/100006436Celgene, Stemline Therapeutics, 10.13039/100017655Incyte, 10.13039/100004336Novartis, Mustang Bio, 10.13039/100016545Roche Diagnostics, and LFB; grants/funding from Affymetrix and Sager Strong Foundation; and board memberships (noncompensated) with Dan’s House of Hope (board of directors) and HemOnc Times/Oncology Times (board member, editor-in-chief). T.C.P.S. reports consulting/advisory role with Novartis, GlaxoSmithKline (GSK), Bristol Myers Squibb (BMS), Rain Oncology, and AbbVie; and received research funding from Imago Biosciences/10.13039/100004334Merck and CellCentric. F.P. reports consulting role with, and honoraria from, AbbVie, Amgen, AOP, BMS/Celgene, Novartis, CTI, GSK, Grifols, Karyopharm, MorphoSys, Sierra Oncology, and Sobi. C.H. reports consulting/advisory role with Promedior, 10.13039/100006436Celgene, 10.13039/501100015086AOP Orphan Pharmaceuticals, 10.13039/100022781Galectin Therapeutics, GSK, Keros, and AbbVie; honoraria from 10.13039/100006436Celgene, 10.13039/100004336Novartis, 10.13039/100017577CTI BioPharma Corp, Geron, 10.13039/501100015086AOP Orphan Pharmaceuticals, BMS, and Constellation Pharmaceuticals; speaker's bureau membership with 10.13039/100004336Novartis, 10.13039/100017577CTI BioPharma Corp, 10.13039/100017655Incyte, 10.13039/100006436Celgene, and 10.13039/100005565Janssen Oncology; and received research funding from 10.13039/100004336Novartis (to institution), 10.13039/100006436Celgene (to institution), and 10.13039/100016480Constellation Pharmaceuticals (to institution). R.S.K. reports speaker's bureau membership with AbbVie, BMS, and Jazz Pharmaceuticals; reports consulting/advisory role with Acceleron, Agios, BMS, Daiichi Sankyo, Inc., Geron, Gilead, and Novartis; and reports stock and other ownership interests with AbbVie. A.P. reports consulting/advisory role with AbbVie, GSK, Novartis, Kartos Therapeutics, and CTI. R.M.A.D. reports consulting/advisory role with Novartis, Incyte, GSK, and BMS; and reports speaker's bureau membership with Astellas and Novartis. D.L. reports consulting/advisory role with AbbVie, Takeda, and Novartis. J.P., Q.Q., J.H., Y.F., and A.R.P. are AbbVie employees and may hold stock. J.S.G. reports consulting/advisory role with AbbVie, BMS, Servier, and Genentech; and received research funding (institutional) from AbbVie, Genentech, Pfizer, Prelude, and New Wave. A.T. reports research funding; Chugai Pharmaceutical, Astellas Pharma, Eisai, Otsuka Pharmaceutical, Ono Pharmaceutical, Kyowa Kirin, Shionogi, Sumitomo Dainippon Pharma, Taiho Pharmaceutical, Takeda Pharmaceutical, Teijin, Nippon Shinyaku, Nihon Pharmaceutical, Pfizer Japan, Mochida Pharmaceutical, Yakult Honsha, and Perseus Proteomics. Lecture fee; AstraZeneca, Chugai Pharmaceutical, AbbVie GK, Genmab, Kyowa Kirin, Eisai, Takeda Pharmaceutical, Astellas Pharma, Nippon Shinyaku, Janssen Pharmaceutical, Zenyaku Kogyo, Bristol Myers Squibb, and SymBio Pharmaceutical.

## References

[1] de Freitas RM, da Costa Maranduba CM (2015). Myeloproliferative neoplasms and the JAK/STAT signaling pathway: an overview. Rev Bras Hematol Hemoter.

[2] Tognon R, Gasparotto EP, Neves RP (2012). Deregulation of apoptosis-related genes is associated with PRV1 overexpression and JAK2 V617F allele burden in essential thrombocythemia and myelofibrosis. J Hematol Oncol.

[3] Tefferi A (2018). Primary myelofibrosis: 2019 update on diagnosis, risk-stratification and management. Am J Hematol.

[4] Tefferi A, Cervantes F, Mesa R (2013). Revised response criteria for myelofibrosis: International Working Group-Myeloproliferative Neoplasms Research and Treatment (IWG-MRT) and European LeukemiaNet (ELN) consensus report. Blood.

[5] Mughal TI, Vaddi K, Sarlis NJ, Verstovsek S (2014). Myelofibrosis-associated complications: pathogenesis, clinical manifestations, and effects on outcomes. Int J Gen Med.

[6] Vannucchi AM, Barbui T, Cervantes F (2015). Philadelphia chromosome-negative chronic myeloproliferative neoplasms: ESMO Clinical Practice Guidelines for diagnosis, treatment and follow-up. Ann Oncol.

[7] (2023). Jakafi (ruxolitinib) tablets. Prescribing information.

[8] US Food and Drug Administration. FDA Approves Fedratinib for Myelofibrosis. 2019. https://www.fda.gov/drugs/resources-information-approved-drugs/fda-approves-fedratinib-myelofibrosis.

[9] US Food and Drug Administration. FDA Approves Drug for Adults With Rare Form of Bone Marrow Disorder. 2022. https://www.fda.gov/drugs/news-events-human-drugs/fda-approves-drug-adults-rare-form-bone-marrow-disorder.

[10] GSK. Ojjaara (Mmelotinib) Approved in the US as the First and Only Treatment Indicated for Myelofibrosis Patients With Anaemia. 2023. https://www.gsk.com/en-gb/media/press-releases/ojjaara-momelotinib-approved-in-the-us-as-the-first-and-only-treatment-indicated-for-myelofibrosis-patients-with-anaemia/.

[11] Pemmaraju N, Verstovsek S, Mesa R (2022). Defining disease modification in myelofibrosis in the era of targeted therapy. Cancer.

[12] Harrison C, Kiladjian JJ, Al-Ali HK (2012). JAK inhibition with ruxolitinib versus best available therapy for myelofibrosis. N Engl J Med.

[13] Harrison CN, Vannucchi AM, Kiladjian JJ (2016). Long-term findings from COMFORT-II, a phase 3 study of ruxolitinib vs best available therapy for myelofibrosis. Leukemia.

[14] Pardanani A, Tefferi A (2018). How I treat myelofibrosis after failure of JAK inhibitors. Blood.

[15] Pardanani A, Tefferi A (2014). Definition and management of ruxolitinib treatment failure in myelofibrosis. Blood Cancer J.

[16] Guo J, Roberts L, Chen Z, Merta PJ, Glaser KB, Shah OJ (2015). JAK2V617F drives Mcl-1 expression and sensitizes hematologic cell lines to dual inhibition of JAK2 and Bcl-xL. PLoS One.

[17] Petiti J, Lo Iacono M, Rosso V (2020). Bcl-xL represents a therapeutic target in Philadelphia negative myeloproliferative neoplasms. J Cell Mol Med.

[18] Refici M, Yang Z, Riehm J, Phillips D, Souers A, Harb J (2022). BCL2A1 is expressed in myelofibrosis specimens and JAK2-mutated UKE-1 cells, yet does not inhibit synergistic cell killing by BCL-XL inhibitor navitoclax plus JAK1/2 inhibitors, including ruxolitinib [abstract]. Cancer Res.

[19] Tse C, Shoemaker AR, Adickes J (2008). ABT-263: a potent and orally bioavailable Bcl-2 family inhibitor. Cancer Res.

[20] Waibel M, Solomon VS, Knight DA (2013). Combined targeting of JAK2 and Bcl-2/Bcl-xL to cure mutant JAK2-driven malignancies and overcome acquired resistance to JAK2 inhibitors. Cell Rep.

[21] Harrison CN, Garcia JS, Somervaille TCP (2022). Addition of navitoclax to ongoing ruxolitinib therapy for patients with myelofibrosis with progression or suboptimal response: phase II safety and efficacy. J Clin Oncol.

[22] Branch SK (2005). Guidelines from the International Conference on Harmonisation (ICH). J Pharm Biomed Anal.

[23] Mesa RA, Kantarjian H, Tefferi A (2011). Evaluating the serial use of the Myelofibrosis Symptom Assessment Form for measuring symptomatic improvement: performance in 87 myelofibrosis patients on a JAK1 and JAK2 inhibitor (INCB018424) clinical trial. Cancer.

[24] Mesa RA, Schwager S, Radia D (2009). The Myelofibrosis Symptom Assessment Form (MFSAF): an evidence-based brief inventory to measure quality of life and symptomatic response to treatment in myelofibrosis. Leuk Res.

[25] Passamonti F, Cervantes F, Vannucchi AM (2010). A dynamic prognostic model to predict survival in primary myelofibrosis: a study by the IWG-MRT (International Working Group for Myeloproliferative Neoplasms Research and Treatment). Blood.

[26] Thiele J, Kvasnicka HM, Facchetti F, Franco V, van der Walt J, Orazi A (2005). European Consensus on grading bone marrow fibrosis and assessment of cellularity. Haematologica.

[27] Common Terminology Criteria for Adverse Events v4.03. National Cancer Institute 2010. https://ctep.cancer.gov/protocoldevelopment/electronic_applications/ctc.htm#ctc_40.

[28] Patel KP, Newberry KJ, Luthra R (2015). Correlation of mutation profile and response in patients with myelofibrosis treated with ruxolitinib. Blood.

[29] Spiegel JY, McNamara C, Kennedy JA (2017). Impact of genomic alterations on outcomes in myelofibrosis patients undergoing JAK1/2 inhibitor therapy. Blood Adv.

[30] Pemmaraju N, Garcia JS, Perkins A (2023). New era for myelofibrosis treatment with novel agents beyond Janus kinase-inhibitor monotherapy: focus on clinical development of BCL-X(L) /BCL-2 inhibition with navitoclax. Cancer.

[31] Mascarenhas J, Hoffman R, Talpaz M (2018). Pacritinib vs best available therapy, including ruxolitinib, in patients with myelofibrosis: a randomized clinical trial. JAMA Oncol.

[32] Harrison CN, Vannucchi AM, Platzbecker U (2018). Momelotinib versus best available therapy in patients with myelofibrosis previously treated with ruxolitinib (SIMPLIFY 2): a randomised, open-label, phase 3 trial. Lancet Haematol.

[33] Verstovsek S, Vannucchi A, Gerds A (2022). S195: MOMENTUM: phase 3 randomized study of momelotinib (MMB) versus danazol (DAN) in symptomatic and anemic myelofibrosis (MF) patients previously treated with a JAK inhibitor. HemaSphere.

[34] Pemmaraju N, Garcia JS, Potluri J (2022). Addition of navitoclax to ongoing ruxolitinib treatment in patients with myelofibrosis (REFINE): a post-hoc analysis of molecular biomarkers in a phase 2 study. Lancet Haematol.

[35] Pemmaraju N, Mead AJ, Somervaille TC (2023). Transform-1: a randomized, double-blind, placebo-controlled, multicenter, international phase 3 study of navitoclax in combination with ruxolitinib versus ruxolitinib plus placebo in patients with untreated myelofibrosis. Blood.

[36] Kuykendall AT, Shah S, Talati C (2018). Between a rux and a hard place: evaluating salvage treatment and outcomes in myelofibrosis after ruxolitinib discontinuation. Ann Hematol.

[37] Palandri F, Breccia M, Bonifacio M (2020). Life after ruxolitinib: reasons for discontinuation, impact of disease phase, and outcomes in 218 patients with myelofibrosis. Cancer.

[38] Verstovsek S, Mesa RA, Gotlib J (2012). A double-blind, placebo-controlled trial of ruxolitinib for myelofibrosis. N Engl J Med.

[39] Verstovsek S, Mesa RA, Gotlib J (2015). Efficacy, safety, and survival with ruxolitinib in patients with myelofibrosis: results of a median 3-year follow-up of COMFORT-I. Haematologica.

[40] Harrison CN, Schaap N, Vannucchi AM (2020). Fedratinib in patients with myelofibrosis previously treated with ruxolitinib: an updated analysis of the JAKARTA2 study using stringent criteria for ruxolitinib failure. Am J Hematol.

[41] Newberry KJ, Patel K, Masarova L (2017). Clonal evolution and outcomes in myelofibrosis after ruxolitinib discontinuation. Blood.

[42] Dilley K, Harb J, Jalaluddin M, Hutti JE, Potluri J (2020). A phase 3, open-label, randomized study evaluating the efficacy and safety of navitoclax plus ruxolitinib versus best available therapy in patients with relapsed/refractory myelofibrosis (TRANSFORM-2). Blood.

