# Supplementary material for: Addition of navitoclax to ruxolitinib for patients with myelofibrosis with progression or suboptimal response
Source: Blood Neoplasia. 2024 Nov 2;2(1):100056. doi: 10.1016/j.bneo.2024.100056 (PMC12082167; doi:10.1016/j.bneo.2024.100056)
Supplement: Supplemental Methods, Table, and Figures [file BNEO_NEO-2024-000338-mmc1.pdf]

SUPPLEMENTAL MATERIALS

Supplemental Methods

Inclusion criteria for Cohort 1a only

Patient must have received ruxolitinib therapy for ≥12 weeks and currently be on a stable dose of ≥10 mg twice daily of ruxolitinib for ≥8 weeks prior to the 1st dose of navitoclax *(Note: Subjects with ruxolitinib dose reductions within 8 weeks prior to study enrollment may be considered on a stable dose if stable at that decreased dose of ruxolitinib for ≥2 weeks prior to the 1st dose of navitoclax. If the dose reduction was due to thrombocytopenia, the platelets must be confirmed to be stable by a repeat laboratory test)*

Inclusion criteria for Cohort 1b only

1. Patients must have received treatment with ruxolitinib and met at least one of the following criteria:
    a. Prior or current treatment with ruxolitinib for ≥24 weeks with suboptimal response – per protocol: lack of efficacy defined as a lack of spleen response (refractory) or a loss of spleen or symptom response (relapsed)
    b. Prior or current treatment with ruxolitinib for <24 weeks with documented disease progression while on ruxolitinib
    c. Prior or current treatment with ruxolitinib for ≥28 days with intolerance defined as new red blood cell transfusion requirement (at least 2 units/month for 2 months) while receiving a total daily ruxolitinib dose of ≥30 mg but unable to reduce dose further due to lack of efficacy
2. Patients who are receiving ruxolitinib at the time of screening, must currently be on a stable dose ≥10 mg twice daily of ruxolitinib for ≥4 weeks prior to the first dose of navitoclax (*Note: Patients with ruxolitinib dose reductions within 4 weeks prior to study enrollment are*

considered to be on a stable dose if dose of ruxolitinib is unchanged for ≥2 weeks prior to day 1 of navitoclax. If the dose reduction was due to thrombocytopenia, platelet counts must be confirmed to be stable by a repeat laboratory test)

3. Patients must not have received treatment with a bromodomain and extra terminal inhibitor or an alternate Janus kinase–2 inhibitor other than ruxolitinib
4. Patients should have at least 2 symptoms, each with a score ≥3 or a total score of ≥12, as measured by the Myelofibrosis Symptom Assessment Form v4.0

Relative dose intensity

The relative dose intensity (RDI) was calculated as the ratio of the dose intensity administered to the dose intensity planned (PDI), with dose intensity being the total amount of drug delivered or total amount of the planned doses over the total time duration of treatment. In the PDI calculations for navitoclax, the maximum dose attained in first 8 weeks of treatment due to the ramp-up in Cohort 1a, and 100 mg or 200 mg starting doses based on baseline platelet count in Cohort 1b were considered as the planned navitoclax doses. In RDI calculations for ruxolitinib, the protocol-specified minimum stable ruxolitinib dose of 10 mg twice a day (20 mg total daily dose) regardless of the actual dose prior to study start, was considered the planned dose.

Statistical methods

A sample size of 34 for Cohort 1a and 70 for Cohort 1b was estimated to provide a percentage point estimate of 47.06 and 45.71 for ≥35% spleen volume reduction (SVR_{35}) at Week 24, with exact 95% confidence interval (CI) within 17.55 and 12.16 percentage points from the point estimate under various assumptions about the true SVR_{35} rate, respectively. Also, if true probability of experiencing a serious adverse event (SAE) due to the study drug was 10%, then the probability of observing at least one SAE in 34 subjects and 70 subjects was more than 97% and more than 99% in Cohorts 1a

and 1b, respectively. Therefore, from safety assessment prospective the proposed sample sizes were adequate.

Continuous demographics and baseline characteristics variables, changes in bone marrow fibrosis grade, and safety data were summarized using descriptive statistics. SVR_{35} and ≥50% reduction in total symptom score (TSS_{50}) were calculated as the proportion of patients who achieved SVR_{35} or TSS_{50} at Week 24, and the corresponding 95% CI derived using the Clopper-Pearson method. Time-to-event endpoints were estimated using Kaplan-Meier methodology. These analyses were conducted using SAS version 9.4 (SAS Institute, Inc, Cary, NC) or later under the UNIX operating system.

Safety analyses were performed for all subjects that received ≥1 dose of navitoclax unless otherwise indicated. For the study as a whole, AEs were evaluated by the National Cancer Institute Common Terminology Criteria for Adverse Events v. 4.03. Safety was assessed by evaluating duration of exposure to study drug, adverse events, SAEs, deaths, and changes in laboratory tests and vital sign parameters.

Supplemental Results

Dose intensity

In total, 26 (21%) patients reached the maximum navitoclax dose of 300 mg once a day. Most patients (n=71 [57%]) reached a maximum dose of 200 mg once a day. About two-thirds of patients who completed 24 weeks of treatment in Cohort 1a (n/N = 18/28) and patients who received 200 mg starting dose in Cohort 1b (n/N = 29/43) maintained a dose of ≥100 mg navitoclax through Week 24 of the study. Median dose in Cohort 1a at Week 24 was 200 mg, and 150 mg for patients in Cohort 1b who received 200 mg as a starting dose.

Supplemental Tables and Figures

Supplemental Table 1. Summary of efficacy assessments

Endpoint	Cohorts 1a and 1b (N=125)
**SVR_{35}** (Week 24)	29/125 (23) [16.1, 31.6]
**SVR_{35}** (any time)	49/125 (39) [30.6, 48.3]
**SVR_{35} by subgroups** (any time)	
Age ≥65 years	35/83 (42) [31.4, 53.5]
DIPSS Risk- Intermediate-1	14/36 (39) [23.1, 56.5]
DIPSS Risk- Intermediate-2	27/71 (38) [26.8, 50.3]
DIPSS Risk- High	8/17 (47) [23.0, 72.2]
HMR mutations	25/71 (35) [24.2, 47.5]
**TSS_{50}** (Week 24)	30/125 (24) [16.8, 32.5]
**TSS_{50}** (any time)	57/125 (46) [36. 7, 54.8]
**TSS_{50} by subgroups** (any time)	
Age ≥65 years	38/83 (46) [34.8, 57.1]
DIPSS Risk- Intermediate-1	14/36 (39) [23.1, 56.5]
DIPSS Risk- Intermediate-2	39/71 (55) [42.7, 66.8]
DIPSS Risk- High	3/17 (18) [3.8, 43.4]
HMR mutations	32/71 (45) [33.2, 57.3]
**Anemia response***	14/61 (23) [13.2, 35.5]
**BMF grade reduction** (Week 24)[†]	19/80 (24) [15.0, 34.6]
**SVR_{35} and BMF grade reduction ≥1** (Week 24)	8/19 (42) [20.3, 66.5]
**BMF grade reduction** (any time)[†]	42/109 (39) [29.4, 48.3]
**≥20% VAF reduction in MPN mutations[‡]** (Week 24)	25/88 (28) [19.3, 39.0]
**≥20% VAF reduction of MPN mutations in patients with HMR mutations[§]** (Week 24)	16/53 (30) [18.3, 44.3]

Abbreviations: BMF, bone marrow fibrosis; CI, confidence interval; DIPSS, Dynamic International Prognostic Scoring System; HMR, high molecular risk; MPN, myeloproliferative neoplasm; PRBC, packed red blood cell; SVR_{35}, ≥35% spleen volume reduction; TSS, total symptom score; TSS_{50}, ≥50% reduction in TSS.

Data are n/N (%) [95% CI].

*Anemia response requires a hemoglobin improvement of ≥2 g/dL without PRBC transfusion or erythropoietin mimetics within 4 weeks prior to the increase for transfusion-independent patients with <10 g/dL at baseline. For transfusion-dependent patients, anemia response is defined as a continuous 12-week period without PRBC transfusion after the first dose of the study drug, ending on or before 30 days post last dose, initiation of post-study treatment, disease progression, or

death, whichever occurs earlier.
†Includes patients with baseline and post-baseline (Week 24 and at any time) fibrosis grade, and baseline bone marrow grade >0.
‡Defined as mutations in *JAK2*, *CALR*, and *MPL*.
§Defined as mutations in *ASXL1, SRSF2, EZH2, U2AF1 (Q157), IDH1, or IDH2*.
95% CIs derived from the Clopper-Pearson method.

Supplemental Figure 1. Patient disposition. (A) patients who discontinued treatment; (B) patients who discontinued the study.

A

Patients enrolled: N=125

Received ≥1 dose navitoclax + ruxolitinib: N=125

Patients who remained on treatment: n=48

Patients who discontinued navitoclax and ruxolitinib: n=77

Reasons for navitoclax discontinuations: n (%)

- Adverse events: 28 (22)[a]
  - Thrombocytopenia: 9 (7)[b]
  - Diarrhea: 4 (3)
  - Neuroendocrine carcinoma of the skin: 2 (2)
- Withdrew consent: n=5 (4)
- Progressive disease: n=24 (19)
- Disease relapse: n=2 (2)
- Physician decision: n=9 (7)
- COVID-19 infection: n=1 (1)
- Other: n=8 (6)[c]

Reasons for ruxolitinib discontinuations: n (%)

- Adverse events: n=12 (10)[d]
  - Thrombocytopenia: 4 (3)
- Withdrew consent: n=5 (4)
- Progressive disease: n=24 (19)
- Disease relapse: 2 (2)
- Physician decision: n=10 (8)
- COVID-19 infection: n=1 (1)
- Other: n=23 (18)[c]

B

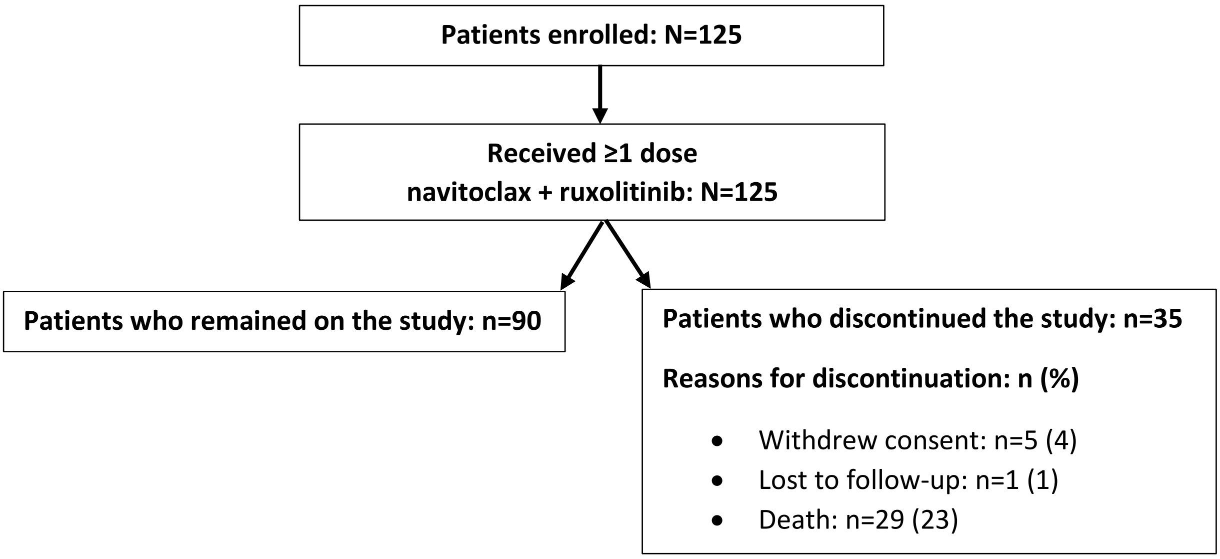

Median duration of study follow-up was 21 months (range 1.6–58.7). The disparity in navitoclax and ruxolitinib reasons for discontinuation is attributed to the combination being discontinued but ruxolitinib being continued as post-study treatment. Primary reasons for discontinuation are shown. Abbreviation: AE, adverse event.

[a]Other AEs leading to navitoclax discontinuation included pneumonia (n=2), deep vein thrombosis, fatigue, hip fracture, fall, tumor lysis syndrome, pleural effusion, device-related infection, cellulitis, increased gamma-glutamyl transferase, increased alkaline phosphatase, and COVID-19 infection (n=1 each).

[b]For the 6 patients who discontinued navitoclax for thrombocytopenia, ruxolitinib was continued as a post-study treatment.

[c]Eight patients discontinued navitoclax (and ruxolitinib) for various reasons, including transplant (n=4), progressive disease, splenectomy, worsened quality of life, and clinical deterioration (n=1 each); the remaining 15/23 patients continued ruxolitinib as post-treatment monotherapy, with the reason for discontinuation noted as “other.”

[d]Other AEs included deep vein thrombosis, pneumonia, hip fracture, fall, diarrhea, increased alanine aminotransferase, COVID-19 infection, and neuroendocrine carcinoma of the skin (n=1 each)

Supplemental Figure 2. Changes in spleen volume over time. Waterfall plot shows maximum percentage change from baseline in spleen volume at any time for Cohorts 1a and 1b.

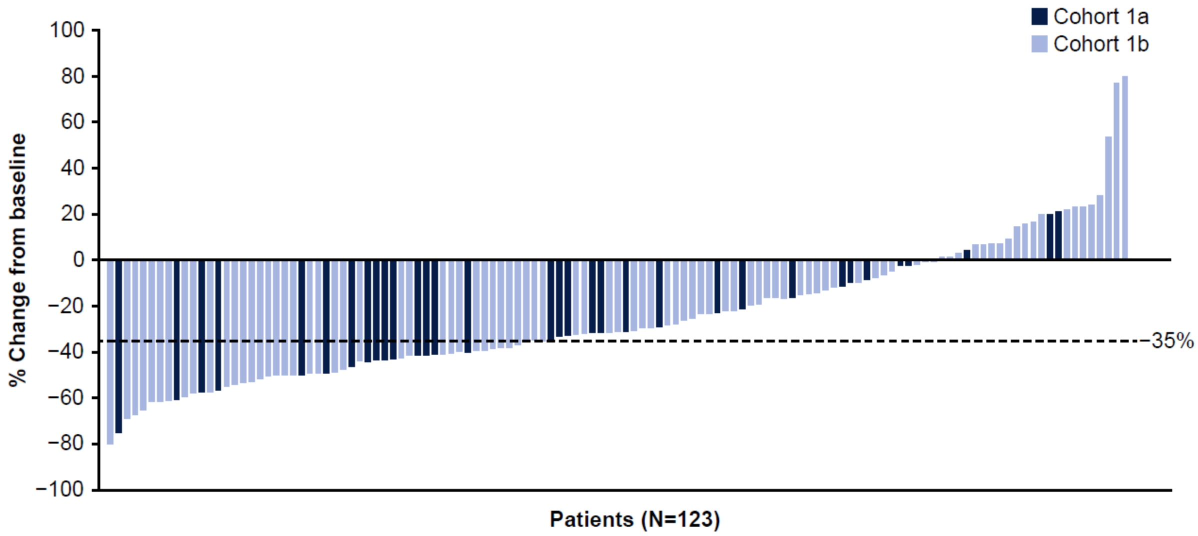

Supplemental Figure 3. Changes in total symptom score over time. Waterfall plot shows maximum percentage change from baseline in TSS at any time for Cohorts 1a and 1b.

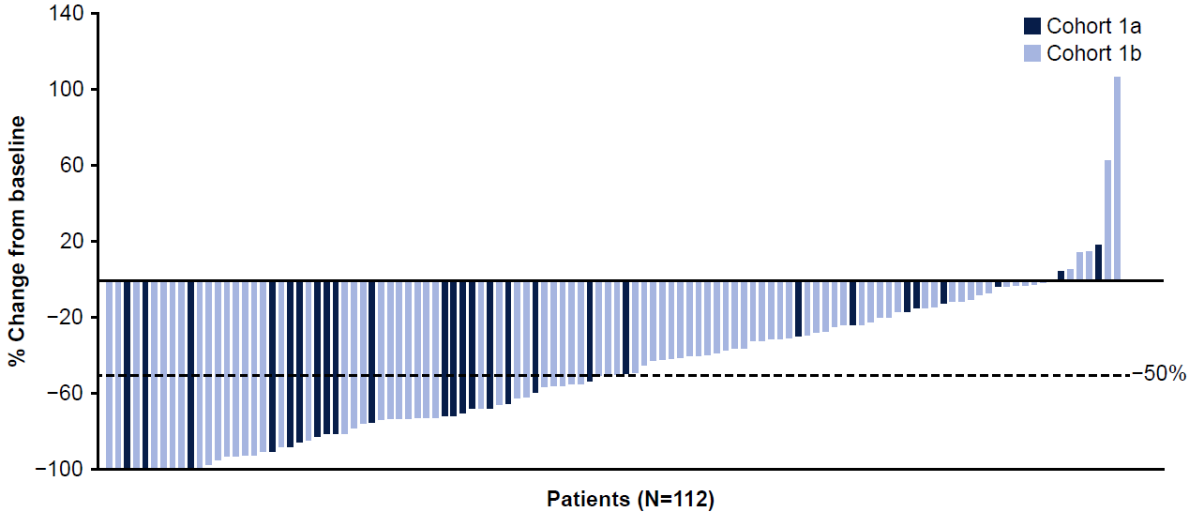

Abbreviation: TSS, total symptom score.

Supplemental Figure 4. Overall survival by bone marrow fibrosis reduction. Kaplan-Meier curve depicts OS by BMF reduction (≥1 grade reduction [yes vs no]) for pooled Cohort 1**.**

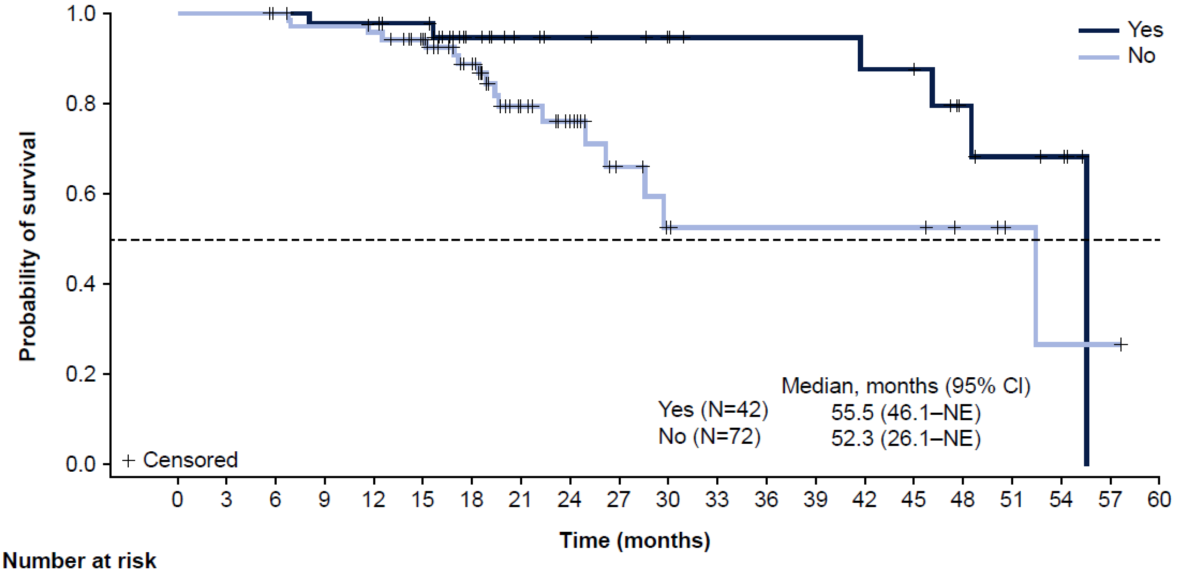

Number at risk

Yes	42	42	41	40	40	38	26	21	18	17	14	13	13	13	12	11	7	5	4	0	
No	72	72	71	68	66	58	44	26	19	11	7	6	6	6	6	6	4	2	1	1	0

Survival estimate % (95% CI)

	Events	Month 6	Month 12	Month 24
Yes (N=42)	6	100.0 (100.0–100.0)	97.6 (83.9–99.7)	94.9 (81.2–98.7)
No (N=72)	17	100.0 (100.0–100.0)	95.7 (87.2–98.6)	76.2 (60.8–86.2)

Abbreviations: BMF, bone marrow fibrosis; CI, confidence interval; NE, not estimable; OS, overall survival.

Supplemental Figure 5. Progression-free survival following treatment with navitoclax and ruxoltinib. Kaplan-Meier curve depicts PFS for pooled Cohort 1.

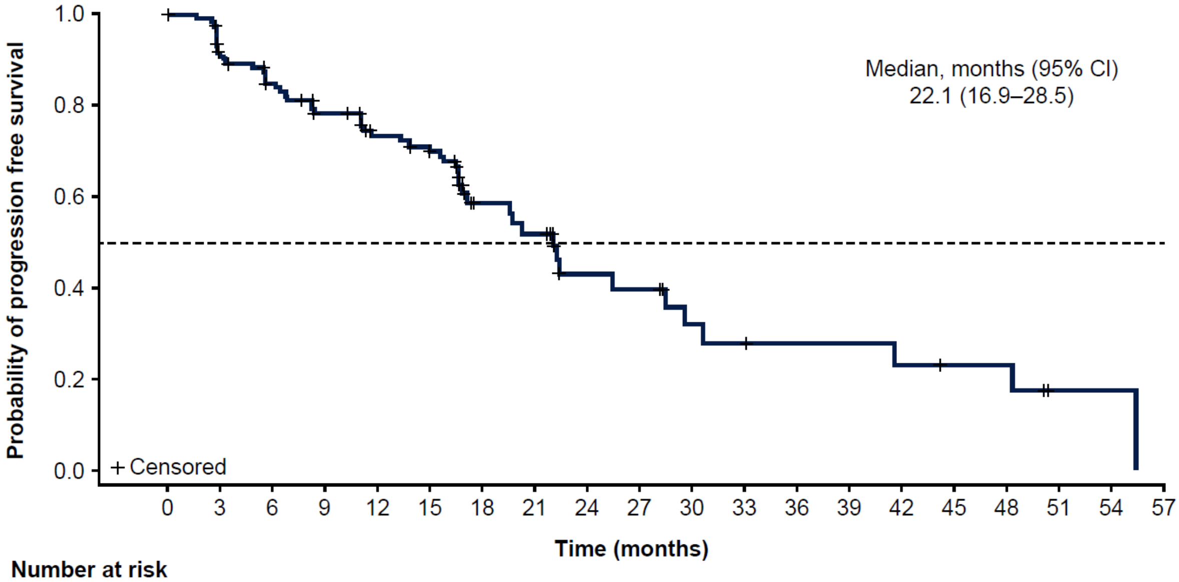

Number at risk

	0	3	6	9	12	15	18	21	24	27	30	33	36	39	42	45	48	51	54	57
Cohort 1	125	107	93	82	66	61	26	23	13	12	8	7	6	6	5	4	4	1	1	0

Survival estimate % (95% CI)

Cohort 1	Events	Month 6	Month 12	Month 24
(N=125)	54	84.9 (77.1–90.2)	73.3 (64.0–80.6)	43.1 (29.6–55.8)

Abbreviations: CI, confidence interval; PFS, progression-free survival.

PFS is defined as time from the first date of study treatment to the date of first documented disease progression/relapse per International Working Group for Myeloproliferative Neoplasms Research and Treatment criteria as determined by the investigator, or death due to any cause, whichever is earlier.
